# Research on Architectural Planning and Landscape Design of Smart City Based on Computational Intelligence

**DOI:** 10.1155/2022/1745593

**Published:** 2022-07-19

**Authors:** Nan Shao

**Affiliations:** Department of Civil Engineering & Architectural, Nanyang Normal University, Nanyang 473061, China

## Abstract

City brain is a complex system, including online center, server network, and system with given algorithm. The core of the city brain is the intelligent system. After putting the urban brain into the intelligent nerve center, on the basis of not changing its original data structure, combining its own characteristics for design and then integrating into application, it can intelligently change the urban management mode. Urban planning leads the development of smart cities on a certain meaning, and smart city planning must have scientific and rational urban planning. The intelligent model is used to make urban planning form a more modern, convenient, and reasonable urban architectural planning. Some influential books on classical architectural theory are the theoretical basis of intelligent urban planning and even the trend and implementation blueprint of how smart cities will develop in the future. In this paper, four algorithms, ant colony algorithm, particle swarm optimization algorithm, genetic algorithm, and improved ant colony algorithm, are proposed to optimize the characteristics of urban architectural planning and landscape design; especially the security research of architecture and landscape characteristics is very important. The improved ant colony algorithm has the shortcoming of insufficient optimization ability in the face of complex path selection. By improving the influencing factors, a new ant colony algorithm is created. The improved ant colony algorithm achieves the best in security features, so it is advocated to use this algorithm for planning and design. The urban form in smart city aims to create a beautiful and comfortable urban environment, improve the competitiveness of cities in the rapid urbanization process, improve the living standards of the public, and shape the image of this beautiful city.

## 1. Introduction

The intelligent model method contributes to the establishment of the model from different aspects such as architectural planning, landscape design, prediction, and uncertainty analysis of urban and smart city development under the development scenario of this era. We propose a description and analysis of smart city governance based on the qualitative method. Contrary to the idea of a centralized smart city imposed by public actors, we notice that Reynes smart city is the result of governance distributed between different poles [1]. Using the data of 274 prefecture-level cities in China from 2004 to 2017, this paper studies the impact of smart city policies on economic green growth and its internal mechanism, which shows that the establishment of smart cities has obviously promoted the green growth of China's economy [2]. Binary phase shift keying (BPSK), quadrature phase shift keying (QPSK), 8-PSK, and 16-PSK are used as various modulation levels. Signal-to-noise ratio (SNR) vs bit error rate (BER) and peak signal-to-noise ratio (PSNR) are used as estimation parameters of received image quality for comparing different versions of OFDM with MRC antenna configuration [3]. By drawing system gram to examine the interaction between these elements, it helps to construct the definition of “smart city,”” then applies the definition to a group of cities, and empirically tests the urban efficiency through data envelopment analysis [4]. By reporting on a brand-new evaluation framework, the global evaluation of the footprint of smart city and community (SCC) projects can also be extended to the cases of smart grid-related projects. The unified smart city evaluation framework is built on three complementary evaluation axes: the first evaluation axis aims to measure the success of an SCC project according to the predefined project-specific target value [5]. This project proposes a music recommendation system based on user emotion detection, automatic calculation, and classification [6]. This work involves the recent research on pedestrian navigation assistance, aiming at finding an alternative to the widely used map-based wheel-by-wheel navigation system in a smart city environment [7]. This paper briefly describes the comprehensive observation scene of environment and humanities in smart cities [8]. Smart city is still a highly related paradigm, which needs further development in order to give full play to its potential and provide strong and flexible solutions. This paper focuses on the Internet of Things as an enabling technology of smart city [9]. In the security framework, we propose a secure video surveillance model, and implement a secure authentication protocol that can resist man-in-the-middle attack (MITM) and replay attack. For the management of video encryption keys, we introduce the Chinese remainder theorem (CRT) based on group key management to provide efficient and secure key update [10]. This paper compares the impacts of primary industry, secondary industry, tertiary industry, and smart industry on Chengdu GDP [11]. Based on the role and participation of public and private actors in (1) capital, (2) asset ownership, and (3) smart city solution operation, a prototype of smart city business model was developed [12]. Using the regression model (*N* = 178), this study shows that the participation difference of public package platform is explained by opinion leaders and political participation, not by community participation and traditional digital inequality [13]. Based on the panel data of 152 prefecture-level cities from 2004 to 2017, this paper uses super-SBM to measure the green land use efficiency [14]. A two-year study of the top ten town halls compared readiness with previous smart city assessment methods: rankings or ISO criteria [15]. Big data analysis in smart cities [16], innovation in information and communication technology (ICT) and the emergence of big data, Internet of Things (IoT), and cloud (BIC) infrastructure have effectively solved the needs of customers and citizens and changed the existing agile city ecosystem [17]. Using the actual data obtained from smart meters installed in Japanese cities, the significance of predicting the temporal and spatial distribution of power demand is demonstrated [18]. Cross-border learning should go beyond the exchange of ideas, suggest facilitators for knowledge transfer, build local policy capacity, encourage cooperative policy transfer, and transition from information-sharing platforms to tool/tool transfer [19]. In this study, the Internet of Things-assisted fog and edge-based smart lamppost are proposed to realize smart infrastructure in smart cities [20]. The reason for formulating an effective urban transformation strategy and transparently selecting independent experts (nonpoliticians) as decision-making and implementation teams is not only the heterogeneity of smart cities in all aspects and the necessary complexity and systematic methods but also the nature of the capabilities and tools required by the concept of smart cities [21]. Reference [22] analyzes in detail the implementation of the concept of smart city in Poland and Ukraine, which belong to the secondary administrative units of neighboring countries, namely, Lublin and Ravi [23]. Taking smart cities as an example, this paper puts forward the construction of automatic hydroponic greenhouses as a model for sustainable generation of urban food. In the construction of this proposal, it is necessary to study the requirements of vegetable planting under hydroponic conditions so as to establish the technology that should be adopted for its automation and identify the basic chemical and climatological requirements in plant biological production [24]. We discussed the potential impact of pandemic on the application of Internet of Things in medical care, smart home, smart building, smart city, transportation, and industrial Internet of Things [25].

## 2. Smart City Feature Planning

### 2.1. Important Characteristics of Urban Brain

High degree of intelligence. The artificial intelligence center of the whole city forms the urban nerve center, an artificial brain system that processes and regulates the information of urban network. If the intelligence of the city is to put a smart hat on the city at any time, then the city brain is to put a host on the city. Locally, wearable smart devices seem to be available, but in essence, they have no ability to operate by themselves and cannot operate jointly with other parts, while the urban brain can coordinate and interact with other parts.

Strong interactivity. Urban brain is a brain-like neuron network related to people, things, and information nodes, and it is also the implementation subject that can solve and deal with various problems and needs of cities. It can bring together data, theory, and algorithm and provide the strongest productivity and production prospect for the information society.

Visualize retina. Visual retina can improve the efficiency ratio from the beginning to the terminal of the urban brain, reduce the algorithm on the line, and make the reflex arc of Internet more accurate and fast. The visual retinal system is based on the two typical application modes of the existing network visual perception system-video acquisition terminal and intelligent terminal. Combining the advantages of the two modes, it can save the storage and bandwidth of urban brain in coding, save the calculation time in online resource consumption, and reduce the delay and improve the accuracy of image feature extraction and analysis.

People-centered. The seventh national census shows that China's population has exceeded 1.4 billion. The construction of population density is the main reference factor of architectural planning, and the core of urban architecture is people. Urban nerve centers can mediate information through the brain. Considering the utilization rate of available resources, the comfort of the surrounding environment to people, air pollution, and other factors, in order to find the optimal solution to meet the necessary needs of human survival and development, a people-centered design concept is comprehensively taken to design a living environment that meets people's own needs.

### 2.2. Urban Architectural Planning of Urban Brain

Optimize urban planning and layout. With the improvement of per capita education level, the number of years of education has increased and the sex ratio has also eased. At the same time, it is accompanied by the large increase of floating population, serious aging, and rapid reduction of rural rate. After the opening of the three-child policy, the population is increasing, but the available resources are limited, so the planning of urban resources has become a top priority, and it is extremely urgent to optimize the layout of urban planning. Through the continuous integration of building information model (BIM), urbanization information model (CIM), and geographic information system (GIS), the urban brain makes full use of visual intelligent technology so that the concept of urban brain can penetrate into smart city planning. On this basis, those were combined with typical theoretical books and materials to determine the design scheme, to greatly optimize the effect of urban planning and design, and to provide basic living security for the follow-up of human life and residence. In this way, according to local conditions, the resource allocation is optimized and the utilization rate of available resources is maximized.

Supervise urban risks and prevent and control risks in advance. Smart cities give alarms and reminders before urban risks come. Taking coastal cities as an example, they follow the goal of “watching natural disasters with one screen and managing the whole city with one network,” uphold the concept of horizontal to edge and vertical to the end, and realize the comprehensive system model of urban risk management with full coverage, all-weather, and whole process. On the basis of this system, it is explored for the first time the construction of an alarm mechanism with normal urban operation environment + risk alarm and the integration of peace and emergency. The intelligent urban management platform can play the role of real-time risk monitoring and unified management of postrisk behavior in the urban planning and operation stage, which can not only effectively prevent and deal with some specific measures of risk coming but also make timely response measures in the face of unexpected situations such as tsunami, earthquake, and other natural disasters.

Sustainable development is an inevitable choice. In urban planning and design, the traditional life concept model has delayed people's pursuit of high-quality life, and the comfort and happiness of people's life have been greatly reduced, and some commercial and trade industries have also been affected to some extent. Infiltrating the concept of smart city into urban architectural planning and landscape optimization design can improve the practicality of planning in an all-round way and make more usable space. With the help of intelligent and visual technology, the coordination of available resources can be more rational, and the overall coordination of resource utilization can improve the overall quality of life of people in urban planning and promote the sustainable development of other industries in a wide range, led by the real estate industry.

Information sharing. In recent years, with the development of Internet and Internet of Things, information sharing has gradually penetrated into all aspects of life. However, urban architectural planning and landscape design are closely related to the future trend of the city and the quality of human life, so it is necessary to fully collect and obtain information from all aspects and consider the influence of each factor. To a certain extent, the traditional architectural planning will affect people's quality of life and cannot keep up with the needs of the development of the times. The urban brain permeates the urban planning, collects information at the same time, and establishes a systematic information online sharing platform, which will help coordinate the communication and mutual assistance performance among personnel in various departments, greatly improve work efficiency, and provide experience and reference blueprint for later urban planning. In this way, information sharing is more thorough, the utilization rate is higher, and the information becomes visual, which can effectively promote the accelerated development of society.

## 3. Programming Algorithm

### 3.1. Principle of Ant Colony Algorithm

If the number of ants on path *X* is  *n*_*x*_ and the number of ants on path *Y* is *n*_*y*_, then the total number of ants *n* is

(1) $$\begin{array}{c} n=n_{x}+n_{y}. \end{array}$$

The probability of choosing path *X* and Path *Y* is

(2) $$\begin{array}{c} P_{x}\left(n\right)=\frac{{\left(n_{x}+k\right)}^{h}}{{\left(n_{x}+k\right)}^{h}+{\left(n_{y}+k\right)}^{h}},\\ P_{y}\left(n\right)=1-P_{x}\left(n\right), \end{array}$$

*h* and *k* adjust to simulate the real environment of ant colony routing.

Modeling

(3) $$\begin{array}{c} \left\{\begin{array}{c} x=\mathrm{mod}\left(i,N\right)-0.5,\\ y=N+0.5-\text{ceil}\left(\frac{i}{N}\right). \end{array}\right.\; \end{array}$$

#### 3.1.1. Probability Planning

Ants have two important factors when searching paths, which are the concentration of information and the heuristic function of distance. At one moment, ants move from one grid to another to form a probability, which is expressed as follows:

(4) $$\begin{array}{c} P_{ij}^{k}\left(t\right)=\left\{\begin{array}{cc} \frac{\tau_{ij}^{a}\left(t\right)\eta_{ij}^{B}\left(t\right)}{\sum_{s\in \text{allowed}}\tau_{ij}^{a}\left(t\right)\eta_{ij}^{B}\left(t\right)}, & s\in \text{allowed},\\ 0, & s\in \text{allowed }. \end{array}\right. \end{array}$$

The distance heuristic function is expressed in the above formula as follows:

(5) $$\begin{array}{c} \eta_{ij}=\frac{1}{d_{ij}}\;,\\ d_{ij}=\sqrt{{\left(x_{i}-x_{j}\right)}^{2}+{\left(y_{i}-y_{j}\right)}^{2}}. \end{array}$$

When the heuristic factor *α* of pheromone concentration is larger, the follow-up ants are more inclined to walk through the nodes they have walked through before, which reduces the diversity of paths and makes the ant colony fall into local optimum. When the heuristic factor *α* of pheromone concentration is small, the feedback mechanism of ant colony will be weakened, which is not conducive to the convergence of the algorithm. When the visibility heuristic factor *β* is large, the randomness of ant selection will decrease; otherwise, it will increase. Therefore, it is necessary to adjust these two parameters in the simulation experiment

#### 3.1.2. Update of Information Elements

The update formula of information elements is as follows:

(6) $$\begin{array}{c} \tau_{ij}\left(t+1\right)=\left(1-\rho \right)\tau_{ij}\left(t\right)+\Delta \tau_{ij},\\ \tau_{ij}\left(t\right)=\sum_{k=1}^{m}\Delta \tau_{ij}^{k}\left(t\right). \end{array}$$

### 3.2. Model Updates

(1) In the Ant-Cycle model, the formula Δ*τ*_*ij*_(*t*) is as follows:
  (7) $$\begin{array}{c} \Delta \tau_{ij}\left(t\right)=\left\{\begin{array}{c} \frac{Q}{L_{k}},\\ 0,\text{otherwise}. \end{array}\right. \end{array}$$
(2) In the Ant-Quanity model, the formula Δ*τ*_*ij*_(*t*) is as follows:
  (8) $$\begin{array}{c} \Delta \tau_{ij}\left(t\right)=\left\{\begin{array}{c} \frac{Q}{d_{ij}}\\ 0,otherwise \end{array}\right. \end{array}$$
(3) In the Ant-Density model, the formula Δ*τ*_*ij*_(*t*) is as follows:

(9) $$\begin{array}{c} \Delta \tau_{ij}\left(t\right)=\left\{\begin{array}{c} Q,\\ 0,\text{otherwise}. \end{array}\right. \end{array}$$

#### 3.2.1. Classical Improvement of Algorithm

The difference between the elite ant algorithm and basic ant colony algorithm lies in the different pheromone updating methods, and its pheromone updating formula is as follows:

(10) $$\begin{array}{c} \tau_{ij}\left(t+1\right)=\rho \tau_{ij}\left(t\right)+\Delta \tau_{ij}\left(t\right)+e\Delta \tau_{ij}^{bs}\left(t\right)\;e=\left(0,1\right). \end{array}$$

The pheromone increment formula of ant sorting algorithm is as follows:

(11) $$\begin{array}{c} \tau_{ij}\left(t+1\right)=\rho \tau_{ij}\left(t\right)+\Delta \tau_{ij}\left(t\right)+e\Delta \tau_{ij}^{bs}\left(t\right),\\ \tau_{ij}\left(t+1\right)=\sum_{k=1}^{w}\Delta \tau_{ij},\\ \Delta \tau_{ij}\left(t\right)=\left\{\begin{array}{c} \frac{\left(v-k\right)Q}{L_{k}},\\ 0,\text{otherwise}. \end{array}\right. \end{array}$$

The max-min ant algorithm is a classical improved ant colony algorithm, which effectively improves the slow convergence speed and is easy to fall into the local optimum of basic ant colony algorithm. The update formula of information elements is as follows:

(12) $$\begin{array}{c} \tau_{ij}\left(t+1\right)=\rho \tau_{ij}\left(t\right)+\Delta \tau_{ij}\left(t\right)+\Delta \tau_{ij}^{bs}\left(t\right). \end{array}$$

When the basic algorithm is used to plan the path, the ant colony has not left pheromones on the path at the initial stage, and the pheromones on the path are scarce at this time, so the feasible path cannot be searched quickly. The new heuristic function is as follows:

(13) $$\begin{array}{c} \eta_{ij}=\frac{1}{{\left(d_{ij}+d_{jE}\right)}^{2}}. \end{array}$$

Considering the influence of volatilization factor on algorithm performance, the improved information element updating strategy in this chapter is as follows:

(14) $$\begin{array}{c} \rho \left(t+1\right)=\frac{T}{T+t}\times \frac{1}{e^{1-p\left(t\right)}}, \end{array}$$

where *T* is the total number of iterations and *t* is the current number of iterations.

The volatilization factor in the basic ant colony algorithm has a very important influence on the performance of the algorithm. If the value is set irrationally, the ant may lose the ability of global search, and the convergence speed will also be affected. *ρ* is always constant in the basic algorithm. If *ρ* is too small, when ants find a better path instead of the optimal path, pheromone volatilization is slow due to the influence of *ρ*, which leads to more pheromone accumulation on this path and attracts ants, so it is difficult for ants to find other better paths, which makes the search results fall into local optimal solution. On the contrary, if the P setting is too large, the pheromone will volatilize at a faster speed, and the residual amount on the path cannot attract ants to search, so it is difficult for ants to choose the next moving grid depending on the pheromone concentration, which reduces the search ability and leads to slow convergence speed.

### 3.3. Ant Colony Algorithm

#### 3.3.1. Modeling

Specific process of initializing population is as follows:

1. Set the parameters needed by the algorithm.
2. Judge whether the grid is continuous, and its judgment method is as follows:

(15) $$\begin{array}{c} \Delta =\text{ max}\left\{abs\left(x_{i+1}-x_{i}\right),abs\left(y_{i+1}-y_{i}\right)\right\}. \end{array}$$

When Δ=1, the two grids are continuous; otherwise, the grid is inserted using the average method, which is calculated by

(16) $$\begin{array}{c} \left\{\begin{array}{c} x_{i}^{\prime }=\mathrm{int}\left[\frac{1}{2}\left(x_{i}+x_{i+1}\right)\right],\\ x_{i}^{\prime }=\mathrm{int}\left[\frac{1}{2}\left(x_{i}+x_{i+1}\right)\right],\\ P_{i}^{\prime }=x_{i}^{\prime }+y_{i}^{\prime }. \end{array}\right. \end{array}$$

#### 3.3.2. Algorithm Improvement

Influencing factors are considered in the fitness function, and the new fitness function is as follows:

(17) $$\begin{array}{c} \text{fit}=\;a\times {\text{fit}}_{1}+b\times {\text{fit}}_{2}, \end{array}$$

where *a* and *b* are their weights, and fit_1_ is the length factor.

*fit*_1_ is the length factor:

(18) $$\begin{array}{c} {\text{fit}}_{1}=\frac{1}{\text{length}}, \end{array}$$

*fit*_2_  is the smoothness factor:

(19) $$\begin{array}{c} {\text{fit}}_{2}=\sum_{i=1}^{\text{end}}\frac{1}{\theta },\\ \theta =\overset{\text{end}-1}{\underset{i=1}{\sum }}\left|{\text{arc}}^{'}\right|\frac{x_{i}-x_{i+1}}{y_{i}-y_{i+1}}\left|-{\text{arc}}^{'}\right|\frac{x_{i}-x_{i+1}}{y_{i}-y_{i+1}}\|. \end{array}$$

The max-min ant algorithm is a classical improved ant colony algorithm, which effectively improves the slow convergence speed and is easy to fall into the local optimum of basic ant colony algorithm. The algorithm mainly consists of three parts. First, like the idea of sorting ant algorithm, the shortest path in the current iterative path is strengthened by pheromone, which improves the convergence speed of the algorithm; second, in order to reduce the influence of pheromone concentration on the search results of the algorithm, the range of pheromone quantity on each path is set; and finally, in order to avoid negative feedback leading to pheromone accumulation, set a maximum value for pheromone.

## 4. Experiment

### 4.1. Simulation Experiment

Set the total number of ant experiments to 50, the maximum iteration times to 100, and carry out four simulation experiments with different iteration times. The simulation results are as shown in Table 1.

**Figure 1:**
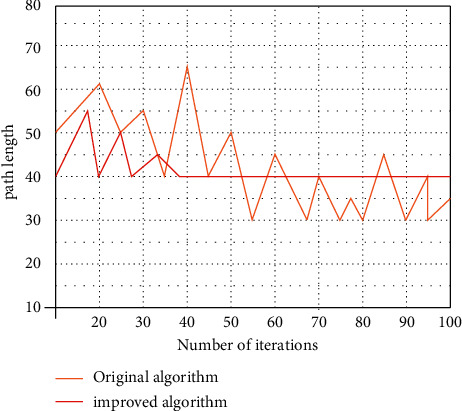
Convergence curve of the algorithm before and after improvement.

When the path becomes complex, the path searched by the basic ant colony algorithm becomes more complicated and tortuous, and it still does not converge when the iteration times reach the maximum, and its optimization ability is extremely unsatisfactory. Its shortcomings are very obvious in a large-scale environment. After improving the ant colony algorithm, we compare the convergence ability of the two, as shown in Figure 1:

The comparison of simulation results shows that the improved algorithm has superiority in optimization effect; that is, it reflects the effectiveness of the algorithm in terms of operation time and shortest path. The improved AG algorithm has obvious advantages in convergence speed and optimal path. It can be well applied in architectural planning and landscape design, and the relative path between buildings can be optimally designed. Smart city landscape design has to bring the best experience to tourists and can bring better time advantage to users visually and temporally.

### 4.2. Model Comparison

**Figure 2:**
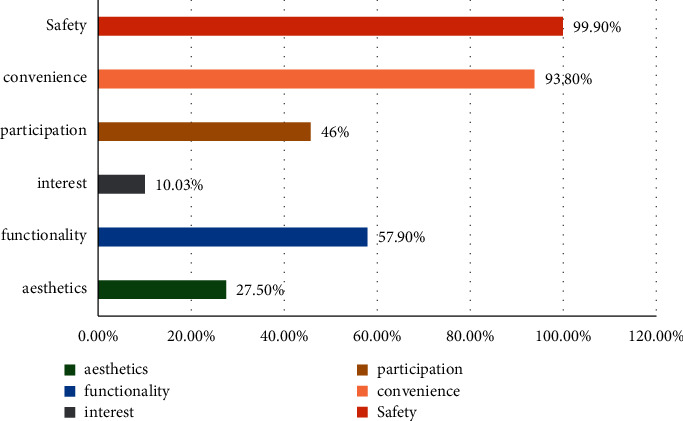
Architectural characteristics.

The urban landscape pattern is the representative of regional characteristics. 500 residents are selected for statistical investigation, and the characteristics of residents' urban architectural planning are counted by the ant colony improvement algorithm. The data are shown in Figure 2.

According to the statistical investigation of residents' desire for urban architectural landscape and the factors of use behavior, private space belongs to personal space demand, semiopen and semiprivate space belong to rest facilities demand, open space belongs to vision demand, and large outdoor square belongs to activity place demand. Using the ant colony improvement algorithm, the relationship between residents' activity mode and behavior mode and urban landscape should be established, as shown in Tables 2, 3, 4 and 5.

**Figure 3:**
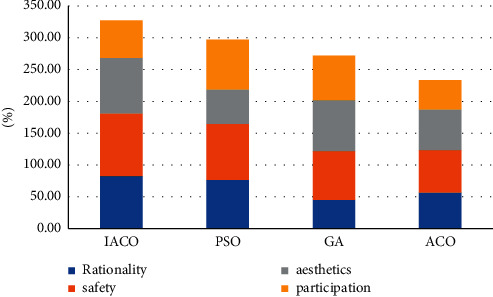
Visual diagram of tree-lined characteristics.

**Figure 4:**
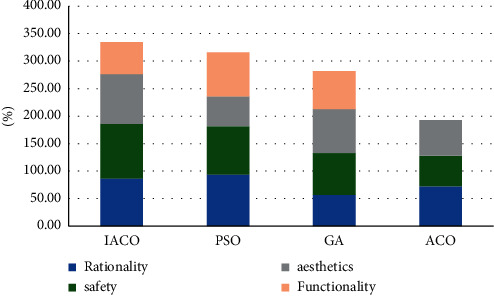
Visual map of lawn characteristics.

**Figure 5:**
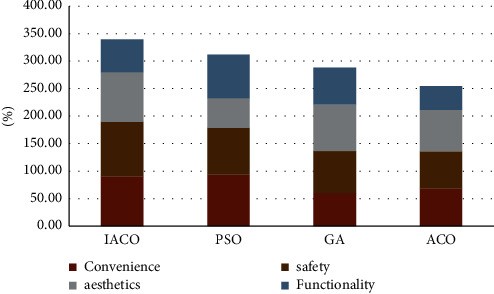
Visual diagram of entertainment square characteristics.

**Figure 6:**
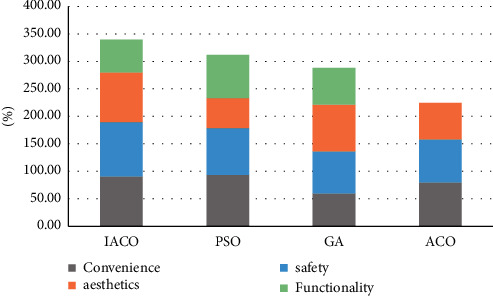
Visual diagram of public toilet characteristics.

Visual statistics of stacked column chart for the data in the above table, as shown in Figure 3:

Visual statistics of stacked column chart for the data in the above table, as shown in Figure 4:

The data in the above table are visually counted by stacking column chart, as shown in Figure 5:

Visual statistics of stacked column chart for the data in the above Table 5, as shown in Figure 6:

### 4.3. Contrast Experiment

**Figure 7:**
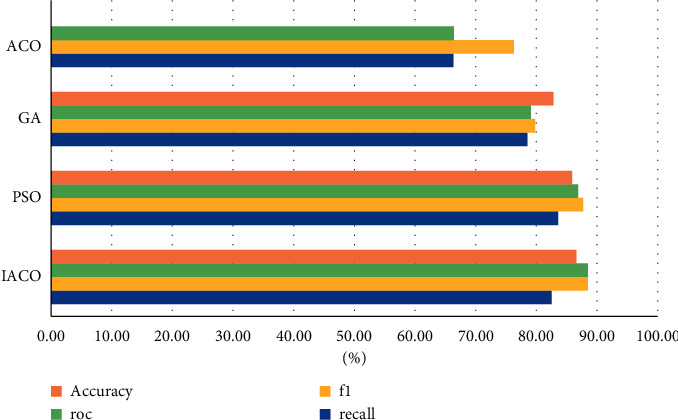
Comprehensive comparison of algorithm performance.

We carry out characteristic statistical analysis on urban buildings and landscapes, respectively, and then compare the performance of four intelligent models, and study the performance of four buildings and landscapes, respectively, to find the best intelligent model. The research data are shown in Tables 6, 7, 8, and 9:

Choose the best value of each performance in the above table to compare the comprehensive performance of the four algorithms, as shown in Figure 7:

## 5. Conclusion

Urban planning leads the intelligent development of cities on a certain meaning, and smart urban planning must have scientific and logical urban planning. For the development of smart cities, smart city planning must have scientific and logical urban planning. The architectural planning and landscape design of smart cities make the urban planning more modern, convenient, and rational. In this paper, intelligent model is used for planning and design, and the research results are as follows:

1. In the simulation experiment, the improved ant colony algorithm before and after the optimal path, average moving times, average calculation time, three parameters comparison, and the improved algorithm is indeed more excellent.
2. According to the convergence curve of the algorithm, it can be seen that under the complex path, the convergence ability of the ant colony algorithm without improvement is poor, but it increases greatly after improvement.
3. Three algorithms are used to study and compare the characteristics of four kinds of architectural landscapes: tree-lined, lawn, entertainment square, and public places.
4. In the comparison experiment, the improved ant colony algorithm is slightly superior to the particle swarm optimization algorithm by comparing the optimal values of the performance comparison table of the three kinds of algorithms mentioned in this paper.

## Figures and Tables

**Table 1:** Results of four simulation experiments.

Parameters	First	Second	Third	Fourth
Optimal path length	31.23	31.28	31.25	31.26
Average number of iterations	31.19	32.03	32.19	30.75
Average operation time	11.06	10.98	11.31	11.24

**Table 2:** Optimal design of landscape tree-lined by each algorithm.

Algorithm	Rationality (%)	Safety (%)	Aesthetics (%)	Participation (%)
ACO	56.8	66.6	64.3	45.8
IACO	82.5	98.5	87.5	58.6
PSO	76.8	87.7	54.4	77.9
GA	45.5	76.3	80.1	69.8

**Table 3:** Optimal design of landscape lawn by each algorithm.

Algorithm	Rationality (%)	Safety (%)	Aesthetics (%)	Functionality (%)
ACO	72.3	55.5	64.8	45.7
IACO	86.8	98.8	90.5	58.6
PSO	93.8	87.7	54.4	79.9
GA	56.5	76.3	80.1	68.8

**Table 4:** Optimal design of architectural entertainment square by each algorithm.

Algorithm	Convenience (%)	Security (%)	Aesthetics (%)	Functionality (%)
IACOAPGA	90.8	98.8	90.5	59.6
ACO	68.6	67.4	75.4	43.2
PSO	93.8	84.7	54.4	78.9
GA	59.9	76.3	85.1	66.8

**Table 5:** Optimal design of public toilets in buildings by each algorithm.

Algorithm	Convenience (%)	Security (%)	Aesthetics (%)	Functionality (%)
IACO	90.6	98.8	90.5	88.6
ACO	80.1	78.1	66.6	67.7
PSO	95.8	87.7	59.4	88.9
GA	66.9	76.3	85.1	69.8

**Table 6:** Performance of each algorithm in landscape tree-lined.

Algorithm	Recall (%)	F1 (%)	Roc (%)	Accuracy (%)
IACO	82.5	88.5	87.5	85.6
ACO	66.3	56.5	60.9	67.3
PSO	80.8	87.7	86.4	85.9
GA	76.5	76.8	78.1	82.8

**Table 7:** Performance of each algorithm in landscape lawn.

Algorithm	Recall (%)	F1 (%)	Roc (%)	Accuracy (%)
IACO	80.5	86.5	84.5	85.8
ACO	56.9	65.2	59.4	66.9
PSO	81.8	85.7	82.4	84.9
GA	77.5	74.8	76.3	80.8

**Table 8:** Performance of each algorithm in architectural entertainment square.

Algorithm	Recall (%)	F1 (%)	Roc (%)	Accuracy (%)
IACO	82.5	85.5	88.5	86.6
ACO	56.8	76.3	66.4	63.5
PSO	80.8	84.7	86.9	85.8
GA	78.5	79.8	78.8	80.8

**Table 9:** Performance of each algorithm in building public toilets.

Algorithm	Recall (%)	F1 (%)	Roc (%)	Accuracy (%)
IACO	82.5	88.5	85.9	85.6
ACO	45.8	65.8	58.4	61.2
PSO	83.6	86.7	86.4	83.9
GA	77.5	76.9	79.1	81.5

## Data Availability

The experimental data used to support the findings of this study are available from the corresponding author upon request.
